# Endocytosis and intracellular processing of BODIPY-sphingomyelin by murine CATH.a neurons^☆^

**DOI:** 10.1016/j.bbalip.2013.08.007

**Published:** 2013-12

**Authors:** Christoph Nusshold, Andreas Uellen, Eva Bernhart, Astrid Hammer, Sabine Damm, Andrea Wintersperger, Helga Reicher, Albin Hermetter, Ernst Malle, Wolfgang Sattler

**Affiliations:** a Institute of Molecular Biology and Biochemistry, Medical University of Graz, Graz, Austria; b Institute of Cell Biology, Histology and Embryology, Medical University of Graz, Graz, Austria; c Institute of Biochemistry, Graz University of Technology, Graz, Austria

**Keywords:** Sphingomyelinase, High density lipoprotein, Caveolar uptake, Endocytosis

## Abstract

Neuronal sphingolipids (SL) play important roles during axonal extension, neurotrophic receptor signaling and neurotransmitter release. Many of these signaling pathways depend on the presence of specialized membrane microdomains termed lipid rafts. Sphingomyelin (SM), one of the main raft constituents, can be formed de novo or supplied from exogenous sources. The present study aimed to characterize fluorescently-labeled SL turnover in a murine neuronal cell line (CATH.a). Our results demonstrate that at 4 °C exogenously added BODIPY-SM accumulates exclusively at the plasma membrane. Treatment of cells with bacterial sphingomyelinase (SMase) and back-exchange experiments revealed that 55–67% of BODIPY-SM resides in the outer leaflet of the plasma membrane. Endocytosis of BODIPY-SM occurs via caveolae with part of internalized BODIPY-fluorescence ending up in the Golgi and the ER. Following endocytosis BODIPY-SM undergoes hydrolysis, a reaction substantially faster than BODIPY-SM synthesis from BODIPY-ceramide. RNAi demonstrated that both, acid (a)SMase and neutral (n)SMases contribute to BODIPY-SM hydrolysis. Finally, high-density lipoprotein (HDL)-associated BODIPY-SM was efficiently taken up by CATH.a cells. Our findings indicate that endocytosis of exogenous SM occurs almost exclusively via caveolin-dependent pathways, that both, a- and nSMases equally contribute to neuronal SM turnover and that HDL-like particles might represent physiological SM carriers/donors in the brain.

## Highlights

• Uptake of BODIPY-SM by murine CATH.a neurons is primarily mediated via caveolar mechanisms.
• Approximately 50% of BODIPY-SM is located in the outer leaflet of the plasma membrane.
• BODIPY-SM is efficiently converted to BODIPY-Cer.
• Both, acidic and neutral sphingomyelinases contribute to BODIPY-SM hydrolysis.
• High density lipoproteins could act as physiological SM delivery system to neurons in the CNS.

## 1 Introduction

Normal brain function depends on a delicately balanced set of remarkably diverse lipids. Within the different cerebral lipid subclasses sphingolipids (SL) take a central role in CNS and neuronal function [1]. The highly divergent group of SL is comprised of sphingosines (Sph), ceramides (Cer), sphingomyelins (SM), and various phosphorylated and complex glycosylated derivates [2]. SL are built of a sphingoid backbone, fatty acid residues (bound via an amide bond) of varying length, and polar head groups with a wide range of complexity [3]. SL are enriched in the CNS, where, in addition to structural roles, SL metabolites act as second messengers that regulate vital signaling events [4–6]. Therefore it is not surprising that dysfunctional SL homeostasis is associated with neurodegenerative diseases [3,7–9].

De novo SL synthesis starts in the ER from palmitoyl CoA and serine to dihydrosphingosine that receives a fatty acid residue to form dihydroceramide, which is then desaturated to form Cer [6]. Cer species are implicated in diverse cellular signaling circuits including apoptosis, growth inhibition, or senescence [2]. Cer is located in a strategic position in SL metabolism [10] and is the immediate precursor for SM synthesis occurring mainly in the trans-Golgi network [2]. To a lesser extent, SM synthesis proceeds at the plasma membrane (PM) via phosphocholine transfer from phosphatidylcholine. These reactions are catalyzed either by SM synthase (SMS)1 or SMS2 [11]. SMS1 is located exclusively to the Golgi apparatus, whereas SMS2 is present at the Golgi and the PM [11,12]. Once synthesized, the majority of SM is transported back to the PM via a vesicular transport mechanism [13]. Like other complex SL, SM is mainly found at the outer leaflet of the PM in specialized microdomains termed lipid rafts [14]. These cholesterol- and SL-enriched microdomains are important PM platforms that compartmentalize cellular processes like e.g. neurotrophic receptor signaling [15] or insertion of glutamate receptors [16]. Of note, SM plays a central role in Golgi vesicle formation and subsequent cargo sorting [17], which might provide an important transport route to dendrites of pyramidal neurons [18]. Furthermore, in the presence of extracellular apoA-I (the major apolipoprotein of high-density lipoprotein; HDL), cells are able to export SM via an ATP-binding cassette transporter A1 (ABCA1)-dependent pathway. During this efflux process SM is one of the lipids exported with highest efficacy to these newly assembled HDL particles [19]. These findings indicate that HDL particles are important in vivo carriers of circulating SM.

In the Salvage pathway a family of sphingomyelinases (SMases) generates phosphocholine (which is further transferred to diacylglycerol) and Cer from SM. This class of enzymes is classified according to their pH optima into acidic- (aSMase), alkaline-, and neutral (nSMase) SMases. Currently four different nSMases that vary in subcellular localization and ion dependency have been described [20]. These enzymes play a central role in brain function since mutations or deficiency in SMases leads to severe neurodegenerative disorders [4]. Hydrolysis of Cer by ceramidases produces another bioactive lipid, Sph, which in turn can be rapidly phosphorylated by sphingosine kinases producing sphingosine-1-phosphate (S1P). The pathways controlling generation of Cer, Sph and S1P have emerged as key pathways regulating the formation and interconversion of these bioactive SL. Sph is a potent effector of SNARE complex assembly thereby facilitating synaptic vesicle fusion and exocytosis [21,22].

Delicately balanced SL metabolism is necessary for neuronal conductivity, viability, and synaptic transmission [23]. Therefore the present in vitro study aimed to i) investigate endocytic pathways (clathrin- and caveolae-dependent) mediating SM uptake, ii) characterize intracellular trafficking and localization of fluorescently-labeled SL derivatives, iii) study the kinetics of SM/Cer interconversion and iv) depict the contribution of SMases to Cer generation from exogenously supplied SM in the murine CATH.a neuronal cell line.

## 2 Materials and methods

### 2.1 Materials

Cell culture supplies were from Gibco (Invitrogen, Vienna, Austria), PAA Laboratories (Linz, Austria), Bartelt (Graz, Austria), and Costar (Vienna). Chlorpromazine, methyl-β-cyclodextrin, genistein, nystatin, chelerythrine chloride, nocodazole, brefeldin A, SMase from *Bacillus cereus*, desipramine, and carbonyl cyanide 4-(trifluoromethoxy)phenylhydrazone (FCCP) were from Sigma (Vienna). *N*-(4,4-difluoro-5,7-dimethyl-4-bora-3a,4a-diaza-*S*-indacene-3-pentanoyl)sphingosyl phosphocholine (BODIPY FL C_5_-SM), *N*-(4,4-difluoro-5,7-dimethyl-4-bora-3a,4a-diaza-*s*-indacene-3-dodecanoyl) sphingosyl phosphocholine (BODIPY FL C_12_-SM), *N*-(4,4-difluoro-5,7-dimethyl-4-bora-3a,4a-diaza-*s*-indacene-3-pentanoyl) sphingosine (BODIPY FL C_5_-Cer = BODIPY-Cer), LysoTracker Blue DND-22, ER-Tracker Blue-White DPX, CellMask PM stain, 1,1′-dioctadecyl-3,3,3′,3′-tetraamethylindocarbocyanine perchlorate (DiI), and HOECHST were from Molecular Probes (Invitrogen, Vienna). Cyanine 3 monoreactive NHS ester(Cy3) was from GE Healthcare. Polyclonal rabbit anti-scavenger receptor class B type I (SR-BI) antibody was a kind gift from Dr. Christian Wadsack (Medical University of Graz, Austria). Horseradish peroxidase (HRP)-labeled secondary goat anti-rabbit IgG was from Sigma. BCA protein assay kit and SuperSignal West Pico chemiluminescent substrate for HRP detection were from Pierce (Thermo Scientific, Waltham, MA, USA). GenMute siRNA transfection reagent for Neuro-2A cells was from SignaGen laboratories (Ijamsville, MD, USA). *N*-[10-(1-pyrenyl)decanoyl]-sphingosyl-1-phosphocholine (PYRENE-SM) was synthesized according to the method described previously [24]. siRNAs were from Qiagen (Hilden, Germany). Precision Red advanced protein assay (Reagent #2) was from Cytoskeleton (Denver, CO, USA). All other chemicals were from Sigma, Roth (Vienna), and Merck (Darmstadt, Germany).

### 2.2 Methods

#### 2.2.1 Cell culture

Catecholaminergic CATH.a cells (ATCC, LGC Standards GmbH, Wesel, Germany) were cultured in poly-l-lysine-coated 75 cm^2^ flasks containing RPMI 1640 medium supplemented with 10% (v/v) horse serum (HS), 5% (v/v) fetal calf serum (FCS), 0.4% (v/v) HEPES buffer, 0.4% (w/v) sodium pyruvate, 0.4% (w/v) l-glutamine, and 100 μg/ml penicillin/streptomycin at 37 °C (5% CO_2_). The split ratio of cells was 1:5 and only passages below 30 were used for experiments.

#### 2.2.2 Laser scanning microscopy (LSM)

Fluorescence microscopy was performed on a Leica SP2 (Leica Lasertechnik GmbH, Heidelberg, Germany). Excitation wavelengths for detection of UV, green, red, and far-red fluorescing compounds were 405, 488, 543, and 647 nm, respectively. Fluorescence emission was recorded at 430–450 nm (UV), 500–535 nm (green), 555–620 nm (red), and 665–750 nm (far-red), respectively.

#### 2.2.3 HPLC conditions

HPLC analysis of fluorescent lipids was performed using a method that is based on the protocol of He et al. [25]. Lipids were dissolved in ethanol and the suspension was transferred into 1.5 ml glass autosampler vials equipped with inserts. BODIPY- and PYRENE-labeled lipids were separated by reversed-phase HPLC (Waters HPLC 2690 Separations Module) on a Kromasil C18 reversed-phase column (150 × 4.6 mm, 5 μm particle size; Altmann Analytik, München, Germany) equipped with the corresponding guard column (5 × 4.6 mm, 5 μm particle size) at a flow rate of 0.7–1 ml/min. BODIPY- and PYRENE-labeled lipids were eluted using the gradients displayed in Tables 1 and 2. A Waters 474 fluorescence detector was set to 505/540 nm or 343/378 nm (Ex/Em) to detect BODIPY- or PYRENE-labeled lipids. BODIPY-C_12_-SM was used as internal standard and the product concentration was calculated according to linear regression analysis, which was established from standard curves of the respective fluorescent lipids (BODIPY-C_5_-SM, BODIPY-Cer, and PYRENE-SM, respectively).

#### 2.2.4 Uptake studies of fluorescent SM by CATH.a cells

##### 2.2.4.1 Quantitative analyses

CATH.a cells were plated on poly-l-lysine-coated 35 mm Culture dishes and grown to approx. 80% confluence. For steady state labeling uptake experiments, cells were incubated with 1 μM (final concentration) BODIPY-SM, PYRENE-SM, or BODIPY-Cer in serum-free culture medium for the indicated time periods at 37 °C in the dark. Cells were then washed two times with ice-cold Hank's buffered salt solution (HBSS), scraped in 500 μl HBSS followed by washing the culture dish with additional 500 μl of HBSS. After centrifugation at 1500 ×*g* for 5 min at 4 °C the supernatant was removed and the cell pellet was stored at − 80 °C until used.

For pulse-chase experiments cells were cooled at 4 °C for 10 min. BODIPY-SM (1 μM, final concentration) was then added to ice-cold serum-free culture medium and cells were pulsed for 30 min at 4 °C (to prevent endocytosis) in the dark to allow insertion of BODIPY-SM into the PM. Following two washing steps with ice-cold HBSS, cells were chased in serum-free culture medium for the indicated time periods at 37 °C in the dark. Cells were then washed two times with ice-cold HBSS, scraped, centrifuged and the cell pellet was stored at − 80 °C until used. Additionally, chase medium was collected and lipids were extracted with 3 ml CHCl_3_/MeOH (2:1, v/v). The CHCl_3_ phase was evaporated under a stream of nitrogen and the dried lipid extracts were stored at − 20 °C until HPLC analysis. Prior to HPLC lipids were dissolved in 60 μl ethanol.

For lipid extraction cells were resuspended in 300 μl sterile water (4 °C) and sonicated for 2 × 15 s on ice. The cell extracts were vortexed vigorously and aliquots of 15 μl were taken for determination of the protein content using the Bradford assay. One milliliter CHCl_3_/MeOH (2:1, v/v) was added to the remaining cell suspension, lipids were extracted, and the dried lipid extracts were stored at − 20 °C until HPLC analysis (lipid extracts were reconstituted in 60 μl ethanol). When cells were labeled with PYRENE-SM lipids were dissolved in 35 μl ethanol prior to HPLC analysis.

##### 2.2.4.2 Fluorescence microscopy

Cells were grown to approx. 60% confluence on poly-l-lysine-coated coverslips before starting the experiments. Pulse-chase uptake studies of BODIPY-SM were carried out as described above except that cells were labeled with 2 μM BODIPY-SM. After the indicated times, cells were washed two times with ice-cold HBSS, mounted, and analyzed by LSM. Where indicated, loosely bound fluorescent BODIPY-SM at the PM was removed by a back-exchange (BE) step [26] by incubating cells with 5% fatty acid-free BSA in ice-cold HBSS (six times for 10 min on ice). In some experiments nuclei were counterstained with HOECHST (5 μg/ml, final concentration) for 10 min at 37 °C before BE. Unlike otherwise noted, BODIPY-C_5_-SM was used throughout all experiments and is designated as BODIPY-SM.

#### 2.2.5 Colocalization experiments

To identify BODIPY-SM containing compartments, CATH.a cells grown to approx. 60% confluence on poly-l-lysine-coated coverslips were incubated with specific markers for lysosomes, ER, Golgi, or the PM, respectively. The cells were pulse-labeled with 2 μM BODIPY-SM as described above. After washing two times with ice-cold HBSS, the cells were chased in the presence of the lysosomal tracker Blue DND-22 (70 nM, final concentration) or the ER selective probe Blue-White DPX (500 nM, final concentration) for 30 min at 37 °C followed by a BE of PM-bound BODIPY-SM as described above. For PM staining, the cells were incubated in the presence of CellMask PM Stain (5 μg/ml, final concentration) for 5 min at 37 °C before pulse labeling with BODIPY-SM (see above). After BE, cells were washed two times with ice-cold HBSS, mounted, and subjected to LSM. In case of PM staining, cells were washed two times with HBSS after pulse labeling with BODIPY-SM and were immediately analyzed by fluorescence microscopy.

To visualize the Golgi apparatus cells were stained with BODIPY-Cer (2 μM, final concentration) in the same way as described for BODIPY-SM. Pretreatment with nocodazole (30 μM, final concentration) or brefeldin A (20 μg/ml, final concentration) was performed for 90 min in serum-free culture medium at 37 °C. All subsequent incubation steps were carried out in the presence of nocodazole or brefeldin A. All incubation and staining steps were carried out in serum-free culture medium in the dark.

#### 2.2.6 Inhibition of clathrin- and caveolae-mediated endocytosis in CATH.a cells

To clarify endocytic mechanisms BODIPY-SM uptake was qualitatively (LSM) and quantitatively (HPLC) analyzed in the presence of inhibitors that interfere with clathrin- or caveolae-mediated uptake. To inhibit clathrin-dependent pathways, a potassium (K^+^) depletion protocol and chlorpromazine (CP) treatment were used. Caveolae-mediated endocytosis was inhibited with the cholesterol depleting/sequestering drugs methyl-β-cyclodextrin (MβCD) and nystatin (NY). The tyrosine kinase inhibitor genistein (GE) causes local disruption of the actin network thereby inhibiting recruitment of dynamin II [27]. Chelerythrine (CHEL) inhibits protein kinase Cα, which is present in caveolae and required for internalization [28]. Cells grown to approx. 80% confluence in poly-l-lysine-coated 6 well plates were washed with HBSS and preincubation with inhibitors was performed as described [26] with slight modifications. (a) *CP treatment*: cells were incubated in the presence of 7.5 μg/ml (final concentration) CP for 30 min at 37 °C. (b) *MβCD treatment*: cells were treated with 10 mg/ml (final concentration) MβCD for 1 h at 37 °C to deplete PM cholesterol. (c) *GE treatment*: cells were incubated in the presence of 200 μM (final concentration) GE for 2 h at 37 °C. (d) *NY treatment*: cells were treated with 30 μg/ml (final concentration) NY for 30 min at 37 °C. (e) *CHEL treatment*: cells were incubated in the presence of 1.5 μM (final concentration) CHEL for 1 h at 37 °C. All preincubation steps were performed in serum-free culture medium followed by pulse labeling with 1 μM BODIPY-SM as described above in the presence of the corresponding inhibitors (exception: MβCD was only present during preincubation of CATH.a cells). (f) *K^+^ depletion*: cells were washed with K^+^-free buffer (20 mM HEPES, 140 mM NaCl, 1 mM CaCl_2_, 1 mM MgCl_2_, 1 mg/ml glucose, pH 7.4) and then incubated in K^+^-free hypotonic buffer (K^+^-free buffer diluted 1:1 with distilled water) for 5 min at 37 °C. Subsequently, cells were washed two times with K^+^-free buffer and incubated in the same buffer for 20 min at 37 °C before pulse labeling with BODIPY-SM which was essentially performed in K^+^-free buffer instead of serum-free culture medium. Control experiments were done in the same way except that all buffer solutions contained 10 mM KCl.

After a 30 min chase at 37 °C cells were washed with ice-cold HBSS and PM-bound BODIPY-SM was subjected to BE using BSA as described above except that BE solutions contained an inhibitor cocktail (5 mM NaN_3_, 50 mM 2-deoxyglucose, 2 mM iodoacetate, 10 μM FCCP; final concentrations) to prevent intracellular trafficking. Essentially all washing and BE steps were performed in the presence of the respective endocytosis inhibitors. Following BE, cells were scraped in ice-cold HBSS as described above and cell pellets were stored at − 80 °C until lipid extraction. For HPLC analysis, dried lipid extracts were dissolved in 60 μl ethanol.

Inhibitor studies for LSM were carried out exactly as described above except that cells (approx. 60% confluent on poly-l-lysine-coated coverslips) were labeled with 2 μM BODIPY-SM. LSM was performed as described above.

#### 2.2.7 *B. cereus* SMase treatment

Cells were grown in poly-l-lysine-coated 35 mm culture dishes to approx. 80% confluence. Pulse labeling with 1 μM BODIPY-SM was performed as described above. After washing cells twice with ice-cold HBSS cells were chased in serum-free culture medium for 1 h to ensure equilibration of the tracer in BODIPY-SM accumulating compartments. Cells were then washed once with HBSS containing 5% (w/v) BSA and once with HBSS alone. Subsequently, cells were treated with bacterial SMase (150 mU/ml, final concentration) at 37 °C in serum-free culture medium for the indicated time periods. Cells were washed and scraped in ice-cold HBSS as described above. Cell pellets were stored at − 80 °C until lipid extraction. For HPLC analysis, dried lipid extracts were dissolved in 60 μl ethanol.

#### 2.2.8 Determination of BODIPY-SM endocytosis

CATH.a cells were grown in poly-l-lysine-coated 35 mm culture dishes to approx. 80% confluence. Pulse labeling with 2 μM BODIPY-SM was performed as described above. After washing twice with ice-cold HBSS cells were chased in serum-free culture medium at 37 °C for the indicated time periods. Subsequently, cells were washed with ice-cold HBSS and BODIPY-SM was subjected to BE from the PM in ice-cold HBSS containing 5% (w/v) BSA and an inhibitor cocktail (see endocytosis inhibition assay) to prevent intracellular trafficking (6 × 10 min on ice). Cells were harvested by scraping in ice-cold HBSS as described above. Cell pellets were stored at − 80 °C until lipid extraction. For HPLC analysis, dried lipid extracts were dissolved in 60 μl ethanol.

BE fractions were pooled and lipids were extracted with CHCl_3_/MeOH (2:1, v/v) over night at 4 °C in the dark on a rotating wheel followed by vortexing for 3 min. Dried lipid extracts were stored at − 20 °C until HPLC analysis for which lipids were dissolved in 500 μl ethanol and aliquots were transferred to inserts-containing HPLC autosampler vials.

#### 2.2.9 SMase assays

SMase activity in CATH.a cells was analyzed using a fluorescence assay as described [24] with some modifications. Cells (approx. 80% confluent in poly-l-lysine-coated 6 well plates) were washed twice with ice-cold HBSS, scraped in the same buffer, and were harvested by centrifugation as described above. The supernatant was removed and the cells were either suspended in 100 μl acid lysis buffer (250 mM sodium acetate, 0.2% Triton X-100, pH 5) for analyzing aSMase activity or in 100 μl neutral lysis buffer (20 mM HEPES, 10 mM MgCl_2_, 0.1 mM Na_3_VO_3_, 0.1 mM Na_2_MoO_4_, 10 mM β-glycerophosphate, 750 μM ATP, 2 mM EDTA, 1 mM phenylmethylsulfonyl fluoride (PMSF), 20 μg/ml aprotinin, 20 μg/ml leupeptin, 0.2% Triton X-100, pH 7.4) to measure Mg^2 +^-dependent nSMase activity. For determinations of Mg^2 +^-independent nSMase activity the cells were lysed in 100 μl neutral lysis buffer without MgCl_2_. The samples were sonicated in a bath-type sonicator for 2 min (on ice) and were then incubated on ice for 1 h (vortexing every 10 min). Cell debris were removed by centrifugation (15,000 *× g*, 4 °C, 10 min) and 10 μl aliquots of the supernatant were used for determination of the protein content which was performed in duplicates according to the Precision Red (Reagent #2) advanced protein assay.

BODIPY-SM was used as a fluorescent substrate to determine SMase activity. BODIPY-SM (1.5 nmol in ethanol) was added per ml acid reaction buffer (250 mM sodium acetate, 1 mM EDTA, pH 5) under vortexing to analyze aSMase activity. Activity of nSMase was determined in neutral reaction buffer (20 mM HEPES, 3.5 mM MgCl_2_, pH 7.4) to analyze Mg^2 +^-dependent nSMase activity. For determinations of Mg^2 +^-independent nSMase activity BODIPY-SM was added to neutral reaction buffer without MgCl_2_. Reaction buffer (150 μl; final substrate concentration was 1.13 μM) was transferred into each well of a 96 well plate and 50 μl of the corresponding lysis buffer containing 15 μg protein was added. The plate was shaken on a rotary shaker (1350 rpm) for 30 s and reaction was allowed to proceed at 37 °C for 45 min in the dark. Reaction was stopped by adding 800 μl CHCl_3_/MeOH (2:1, v/v) and lipids were extracted as described above. Dried lipid extracts were stored at − 20 °C until HPLC analysis for which lipids were dissolved in 250 μl ethanol.

#### 2.2.10 RNAi in CATH.a cells

Transfection of cells (approx. 50% confluent in poly-l-lysine-coated 6 well plates) with siRNA was performed using the GenMute siRNA transfection reagent for Neuro-2A cells according to the manufacturer's suggestions. siRNAs used for aSMase (SMPD1), nSMase1 (SMPD2), and nSMase2 (SMPD3) knockdown were from Qiagen (product numbers: SI02738806 (SMPD1), SI01426971 (SMPD2_1), SI01426978 (SMPD2_2), SI01426985 (SMPD2_3), SI01426992 (SMPD2_4), and SI01427006 (SMPD3), respectively). Concentrations of SMPD siRNA constructs were 250 nM (SMPD1 and SMPD3 silencing) and 150 nM (SMPD2 silencing), respectively. Forty-eight hours post transfection the cells were subjected to analysis of SMase activity, qPCR analysis or pulse-chase experiments, which were performed as described above. For combined siRNA and pharmacological treatment (using the aSMase inhibitor desipramine; Des), SMPD1-silenced cells were incubated in the presence of Des (30 μM, final concentration) in serum-free culture medium for 1.5 h. All following steps were carried out in the presence of Des.

#### 2.2.11 RT-qPCR

Cells were transfected with specific siRNAs, lysed and RNA extracts were collected two days post transfection. Total RNA was isolated using the RNeasy Kit. Aliquots of total RNA (3 μg) were reverse transcribed using SuperScript II Reverse Transcriptase and random hexamer primers according to the manufacturer's protocol (Invitrogen). Real-time PCRs were performed with an Applied Biosystems 7900HT Fast Real Time PCR System, the QuantiFast SYBR Green PCR kit, and QuantiTect Primer Assays. Hypoxanthine phosphoribosyltransferase 1 (HPRT) was used as housekeeping gene. Statistical significance of differences in mRNA expression levels was analyzed using the relative expression software tool (REST©, http://www.gene-quantification.de/rest.html) using a pair-wise fixed reallocation test [29].

#### 2.2.12 HDL isolation and labeling procedures

HDL was prepared by density gradient ultracentrifugation of plasma obtained from normolipemic donors [30]. HDL was recovered from the tubes and a desalted (PD-10 column) aliquot was used for protein determination according to the Bradford assay [31]. For BODIPY-SM/DiI co-labeling 5 nmol BODIPY-SM and 50 nmol DiI both dissolved in DMSO were added to HDL (1 mg protein in 500 μl PBS) under vortexing. The solution was incubated at 37 °C for 2 h in the dark followed by gentle mixing on a rotating wheel for further 6 h at room temperature (RT). To prepare BODIPY-SM/Cy3-labeled HDL 10 nmol BODIPY-SM was added under vortexing. Covalent labeling of the protein moiety of HDL was performed by incubation of HDL (2 mg protein in 1 ml 0.1 M NaHCO_3_, pH 8.3) with 70 nmol monofunctional Cy3 dye dissolved in DMSO for 4 h at RT in the dark under gentle mixing on a rotating wheel. Labeled HDL was desalted and separated from unbound fluorophore using a PD-10 column and PBS as eluent. After filter sterilization (0.45 μm pore size) the protein concentration was determined according to the Bradford assay. Fifty micrograms (final concentration) of fluorescently-labeled HDL per ml serum-free culture medium was used for steady state uptake studies. Competition experiments were performed in the presence of a 40-fold excess of unlabeled HDL. Where indicated, endocytosis was blocked by incubation with hypertonic sucrose (0.4 M, final concentration) in serum-free culture medium at 37 °C for 30 min before incubation with fluorescently-labeled HDL.

For quantitative uptake studies cells were incubated in the presence of BODIPY-SM-labeled HDL (50 μg HDL protein/ml, final concentration). At the indicated time points cellular lipids were extracted and analyzed by HPLC as described above.

#### 2.2.13 Western blot analysis of SR-BI expression

Cells (approx. 80% confluent in 6 well plates) cultured in serum-containing (15%; v/v) or serum-free medium were washed with ice-cold PBS and then scraped in 50 μl lysis buffer (50 mM Tris/HCl, pH 7.4, 1% NP-40, 150 mM NaCl, 1 mM Na_3_VO_4_, 1 mM NaF, 1 mM EDTA, 10 μM PMSF, and 1 μg/ml each aprotinin, leupeptin, and pepstatin). After sonication (2 × 10 s on ice) cell debris was removed by centrifugation (13,000 rpm, 4 °C, 10 min) and the protein content was determined using the BCA assay. Protein lysates were separated by SDS-PAGE (130 V, reducing conditions) and transferred to PVDF membranes (150 mA). Polyclonal rabbit anti-SR-BI antibody (1:1000) was used as primary antibody. Immunoreactive bands were visualized using HRP-conjugated goat anti-rabbit IgG (1:5000) and subsequent SuperSignal West Pico development. Porcine brain microvascular endothelial cell protein lysates served as positive control for SR-BI expression [32].

## 3 Results

### 3.1 BODIPY-SM accumulates at the PM at 4 °C and is internalized at 37 °C

**Fig. 1:**
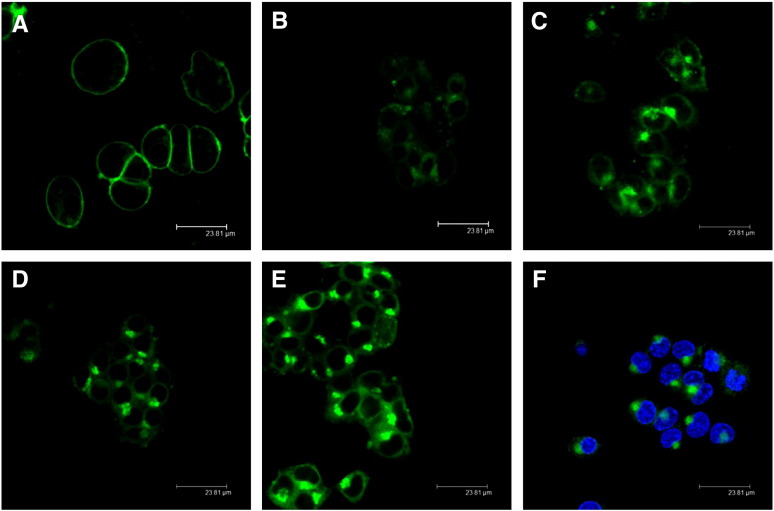
Uptake and subcellular localization studies of fluorescent BODIPY-SM. CATH.a cells were cooled at 4 °C for 10 min and then pulse-labeled with BODIPY-SM (2 μM) in serum-free culture medium for 30 min at 4 °C. Cells were washed with HBSS and chased for (A) 0 min, (B) 5 min, (C) 15 min, (D) 30 min, (E) 60 min, or (F) 240 min at 37 °C. Remaining BODIPY-SM at the PM was removed by BE prior to LSM analysis (B-F). Nuclei in (F) were counterstained with HOECHST (5 μg/ml) for 10 min at 37 °C.

In a first series of experiments time-dependent uptake and subcellular distribution of BODIPY-SM in CATH.a neurons were studied. At 4 °C BODIPY-SM accumulated exclusively at the PM (Fig. 1A). Following removal of PM-localized SM by BE to BSA and a switch to 37 °C fluorescence was observed in punctuated intracellular structures indicative of early recycling endosomes (Fig. 1B). At later time points (15–240 min; Fig. 1C–F) BODIPY-fluorescence is primarily localized at the ER and/or perinuclear regions resembling the Golgi apparatus.

**Fig. 2:**
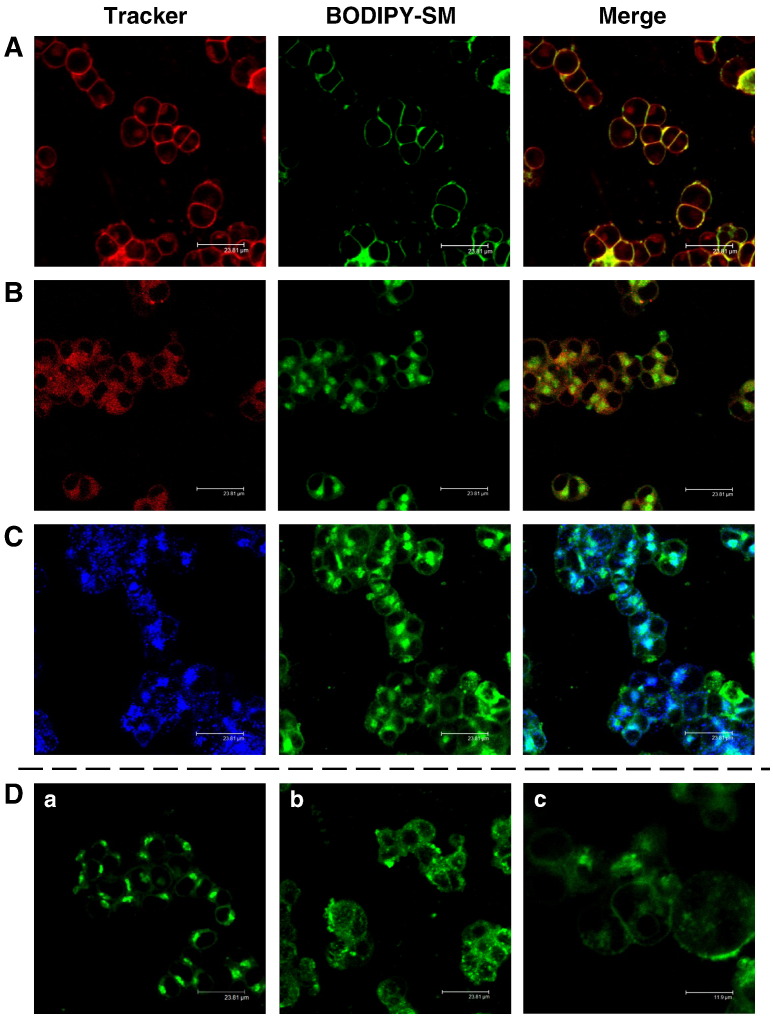
Colocalization of BODIPY-SM with organelle-specific markers. CATH.a cells were cooled at 4 °C for 10 min and then pulse-labeled with BODIPY-SM (2 μM) in serum-free culture medium for 30 min at 4 °C. Cells were stained with (A) CellMask PM stain (5 μg/ml) or were chased for 30 min at 37 °C in the presence of (B) ER-Tracker Blue-White DPX (500 nM) or (C) LysoTracker Blue DND-22 (70 nM). Cells were washed and subjected to BE (B and C). (D) Cells were stained with BODIPY-Cer (2 μM; a) in the same way as described for BODIPY-SM, or pre-incubated with nocodazole (30 μM; b) or brefeldin A (20 μg/ml; c) for 90 min at 37 °C and then pulse-chase-labeled with BODIPY-SM as described above.

To elucidate intracellular localization of BODIPY-SM/Cer in more detail, colocalization experiments with organelle-selective probes were performed. First, colocalization of CellMask PM stain (red) and BODIPY-SM (green) was confirmed at 4 °C (Fig. 2A). Under the experimental conditions applied, part of the PM marker was subject to internalization. At 37 °C partial colocalization of the ER tracker (red) and BODIPY-fluorescence (green) was observed (Fig. 2B), while almost no colocalization of the LysoTracker (blue) with BODIPY-SM (green) was detectable (Fig. 2C). Labeling of cells with the Golgi marker BODIPY-C_5_-Cer resulted in an identical fluorescence pattern as observed with BODIPY-SM (Fig. 2D, a). Pretreatment of cells with the microtubule-disrupting agent nocodazole resulted in accumulation of BODIPY-SM in intracellular punctuate structures (Fig. 2D, b). These ministacks apparently result from Golgi fragmentation due to disruption of the microtubular network. Brefeldin A treatment resulted in PM-associated as well as diffuse intracellular fluorescence, most probably a consequence of BODIPY-SM trapping in an ER-Golgi hybrid compartment (Fig. 2D, c).

### 3.2 Initial pathways of BODIPY-SM endocytosis and turnover in CATH.a neurons

**Fig. 3:**
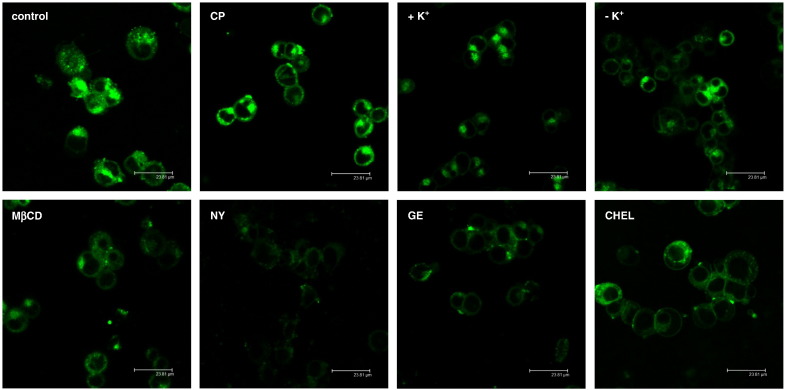
Inhibition of BODIPY-SM endocytosis by pharmacological antagonists. To inhibit clathrin-mediated endocytosis, CATH.a cells were incubated in the absence (control) or presence of chlorpromazine (CP; 7.5 μg/ml; 30 min) or were incubated in the presence (+ K^+^) or absence (− K^+^) of potassium. K^+^ depletion was performed by treatment with K^+^-free hypotonic buffer (control cells (+ K^+^) were treated with hypotonic buffer containing 10 mM KCl). To interfere with caveolar uptake, cells were preincubated in the absence (control) or presence of methyl-β-cyclodextrin (MβCD; 10 mg/ml; 60 min), nystatin (NY; 30 μg/ml; 30 min), genistein (GE; 200 μM; 120 min), or chelerythrine (CHEL; 1.5 μM; 60 min) at 37 °C. Following inhibitor treatment cells were washed with ice-cold HBSS and cooled at 4 °C for 10 min, followed by pulse labeling with BODIPY-SM (2 μM) in serum-free culture medium or the respective K^+^-free/K^+^-containing buffers for 30 min at 4 °C. Cells were washed with HBSS and chased for 30 min. After removal of membrane-bound BODIPY-SM by BE cells were subjected to LSM analysis. All incubation steps were performed in the presence of the inhibitors except for MβCD, which was present only during the preincubation period. All micrographs were recorded with the same laser intensity.

Endocytosis of BODIPY-SM uptake was followed on a qualitative (LSM) and quantitative basis (HPLC). Cells were preincubated in the absence or presence of the respective inhibitors at the indicated concentrations before pulse labeling with BODIPY-SM at 4 °C followed by a 30 min chase at 37 °C. Control cells accumulated the label in perinuclear regions and this pattern was not significantly altered in response to CP treatment or K^+^ depletion (Fig. 3). In contrast, inhibitors of caveolae-dependent endocytosis (MβCD, NY, GE, and CHEL) resulted in lower fluorescence intensity in intracellular compartments. These data suggest that NY (followed by GE, CHEL, and MβCD) inhibited BODIPY-SM uptake with highest efficacy indicating that the majority of BODIPY-SM is internalized via caveolae-mediated pathways.

**Fig. 4:**
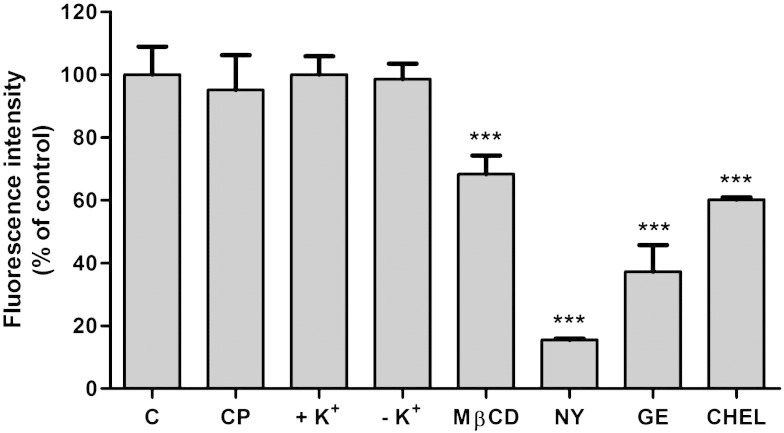
Quantitation of BODIPY-SM endocytosis in the presence of pharmacological antagonists. CATH.a cells were incubated with BODIPY-SM (1 μM) and the corresponding inhibitors as described in Fig. 3. Cells were scraped in ice-cold HBSS and lipids were extracted with CHCl_3_/MeOH. Dried lipids were re-dissolved in ethanol and analyzed by HPLC. Results are expressed as fluorescence intensity as % of control and represent mean ± SD (n = 3) of one representative experiment. ***p < 0.001 (in comparison to controls, ‘C’).

To quantitatively confirm results obtained by fluorescence microscopy CATH.a cells were pretreated with the respective inhibitors and pulse-labeled with BODIPY-SM. Cellular lipids were extracted and analyzed by HPLC. In line with LSM data, CP treatment and K^+^ depletion were without effect on BODIPY-SM uptake (Fig. 4). In contrast, MβCD, NY, GE, and CHEL inhibited BODIPY-SM uptake by 30, 80, 60, and 40%, respectively.

**Fig. 5:**
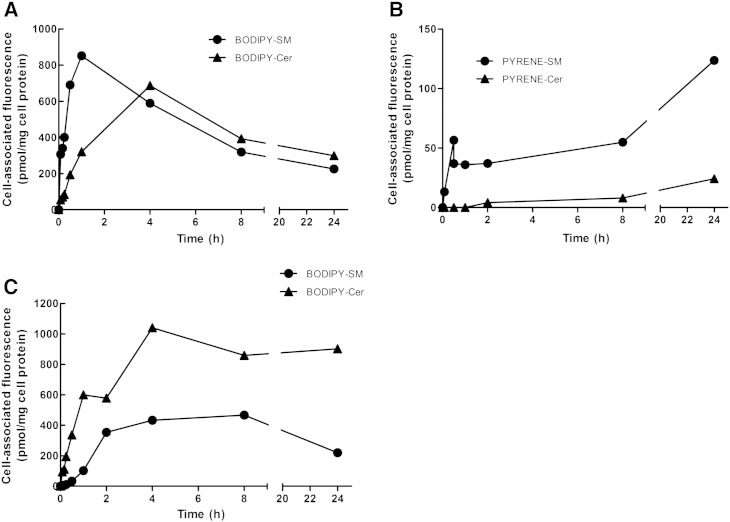
Uptake and metabolism of fluorescent SM and Cer analogs during steady state labeling. CATH.a cells were incubated with (A) BODIPY-SM, (B) PYRENE-SM, or (C) BODIPY-Cer (1 μM each) for the indicated time periods at 37 °C. Cells were washed with HBSS, scraped, and cellular lipids were extracted with CHCl_3_/MeOH, dried, dissolved in ethanol, and analyzed by HPLC. Results shown represent mean values from one representative experiment performed in duplicates.

Having established caveolae-mediated uptake as the major pathway of BODIPY-SM internalization we next investigated intracellular BODIPY-SM hydrolysis to BODIPY-Cer, the first metabolic step in SM remodeling. During these studies we utilized steady state and pulse-chase labeling protocols. To exclude artifacts due to the fluorophore two different fluorescently-labeled SM derivatives were used. During steady state labeling (providing a continuous tracer supply), cells were incubated with BODIPY-SM, PYRENE-SM, or BODIPY-Cer, followed by HPLC analyses of cellular lipids. These experiments revealed that BODIPY-SM hydrolysis is a relatively fast process with BODIPY-Cer generation being detectable already after 5 min (Fig. 5A). BODIPY-SM levels in CATH.a cells peaked after 1 h (850 pmol/mg cell protein) and declined to 225 pmol/mg cell protein after 24 h of incubation. In parallel BODIPY-Cer generation occurred at similar rates as observed for SM accumulation (up to 60 min) and showed highest intracellular concentrations after 4 h of incubation (690 pmol/mg cell protein). Starting from this time point BODIPY-Cer concentrations slightly exceeded the concentrations of cell-associated BODIPY-SM (up to 1.3-fold).

Uptake of PYRENE-labeled SM (carrying a C_10_-acyl residue that more closely resembles physiochemical properties of natural SM) is almost one order of magnitude lower (Fig. 5B) as compared to the BODIPY-labeled analog. PYRENE-SM hydrolysis was less efficient as compared to BODIPY-SM since only 16% of total cell-associated PYRENE fluorescence was converted to PYRENE-Cer after a 24 h steady state labeling experiment.

Next we were interested in the efficacy of BODIPY-SM synthesis from BODIPY-Cer. These data suggest that BODIPY-SM synthesis from BODIPY-Cer is a slower process than BODIPY-SM hydrolysis, reaching a plateau after 4 h (Fig. 5C) where BODIPY-Cer levels still exceed the levels of generated BODIPY-SM up to 2.4-fold.

**Fig. 6:**
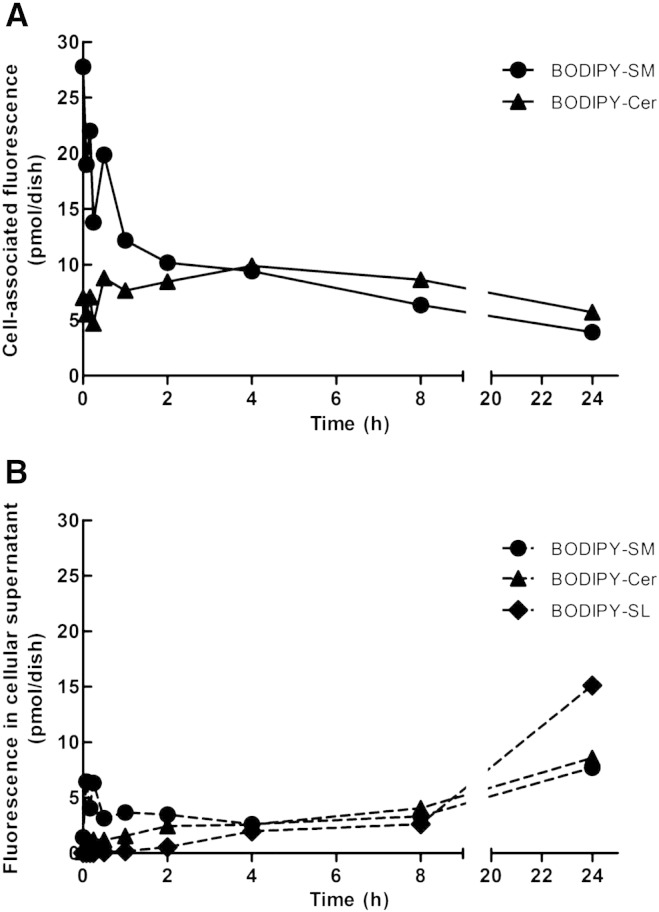
Turnover of BODIPY-SM after pulse labeling. CATH.a cells were cooled at 4 °C for 10 min, pulse-labeled with BODIPY-SM (1 μM) in serum-free culture medium for 30 min at 4 °C, washed, and chased for the indicated time periods at 37 °C. Cells were washed with HBSS, scraped, followed by lipid extraction from (A) cells and (B) the cellular supernatant. Lipid extracts were subjected to HPLC analysis. Results shown represent mean values from one representative experiment performed in duplicates.

To get an indication about the time-dependent metabolism of a defined concentration of PM-located BODIPY-SM in CATH.a cells, we have performed pulse-chase studies and subsequent HPLC analyses of cell and medium lipids. As shown in Fig. 6A cells contained 28 pmol BODIPY-SM and 7 pmol BODIPY-Cer at time zero (indicating partial hydrolysis already at 4 °C). After 24 h cells contained 4 and 6 pmol BODIPY-SM and BODIPY-Cer, respectively, indicating a total loss of 25 pmol cell-associated BODIPY lipids. This loss of cellular lipids was accompanied by a gain in the cellular supernatant containing (after 24 h) 7 pmol BODIPY-SM, 7 pmol BODIPY-Cer, and 15 pmol of a currently unidentified BODIPY-containing lipid (termed BODIPY-SL; Fig. 6B) indicating efficient efflux of BODIPY-labeled SL to the cellular supernatant.

### 3.3 Quantitation of PM-located BODIPY-SM

**Fig. 7:**
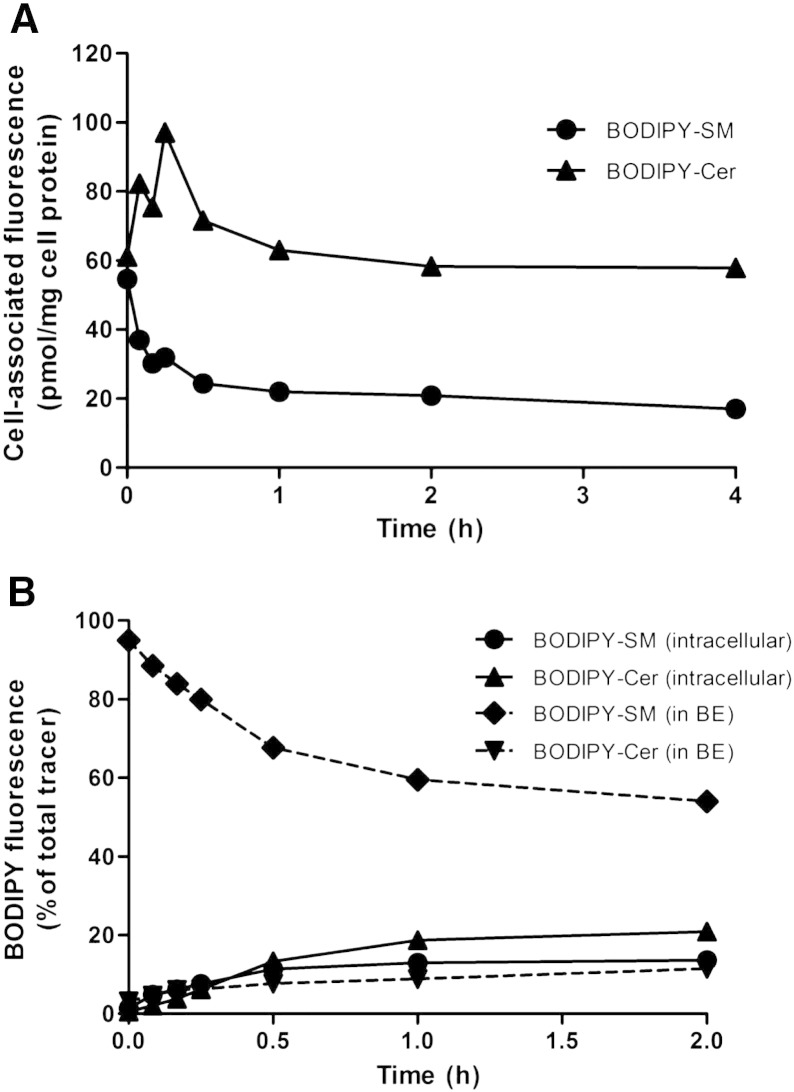
Analysis of PM-located BODIPY-SM. CATH.a cells were cooled at 4 °C for 10 min and pulse-labeled with BODIPY-SM (1 μM for *B. cereus* SMase treatment and 2 μM for the BE protocol) in serum-free culture medium for 30 min at 4 °C. (A) Cells were washed and chased in serum-free culture medium at 37 °C for 60 min to enable BODIPY-SM internalization. Cells were then subjected to *B. cereus* SMase treatment (150 mU/ml) in serum-free culture medium at 37 °C up to 4 h. Cellular lipid extracts were analyzed by HPLC. (B) After pulse labeling cells were washed and chased at 37 °C. At the indicated times cells were subjected to BE at 4 °C followed by BODIPY-SL analysis in the BE fractions and cell extracts by HPLC. Results shown represent mean values from one representative experiment performed in duplicates.

PM-located SM is rapidly metabolized (Fig. 6A), resulting in the generation of Cer (Figs. 5A and 6A), Sph, or S1P [33]. To quantitate the amount of SM residing in the PM of CATH.a neurons two experimental strategies were applied: First, PM-associated BODIPY-SM was subjected to hydrolysis by exogenously added *B. cereus* SMase. Following pulse labeling at 4 °C and a 60 min chase period (37 °C) cells contained 55 and 61 pmol/mg cell protein of BODIPY-SM and BODIPY-Cer, respectively at time zero. Treatment with *B. cereus* SMase reduced the BODIPY-SM concentrations to 17 pmol/mg cell protein, indicating that 67% are accessible to *B. cereus* SMase-mediated hydrolysis (Fig. 7A). Cellular BODIPY-Cer concentrations transiently increased from 61 to 97 pmol/mg cell protein after 15 min of *B. cereus* SMase treatment. Then, concentrations decreased, reaching a plateau after further 45 min (≈ 65 pmol/mg cell protein). This might be a result of Cer efflux to the cellular supernatant and/or Cer conversion to other, more complex SL species.

In a second approach cells were pulse-labeled with BODIPY-SM and then switched to 37 °C to allow internalization. At the indicated times cells were washed and PM-located BODIPY-SM was removed by BE at 4 °C. BODIPY-SM and BODIPY-Cer were quantitated in the BE medium and in cellular lipid extracts (Fig. 7B). Results of these studies revealed that at time zero the majority (92%) of BODIPY-SM was located in the PM (i.e. accessible to BE). After 2 h, 55 and 11% of total fluorescence was recovered as BODIPY-SM and BODIPY-Cer in the BE medium (i.e. PM-associated). The cellular lipid extracts contained 13 and 21% of total fluorescence (BODIPY-SM and BODIPY-Cer), respectively. In summary these experiments indicate that approx. 55 (accessible to BE) to 67% (accessible to *B. cereus* SMase) of BODIPY-SM is located in the outer leaflet of the PM.

### 3.4 Contribution of neuronal SMases to BODIPY-SM hydrolysis

**Fig. 8:**
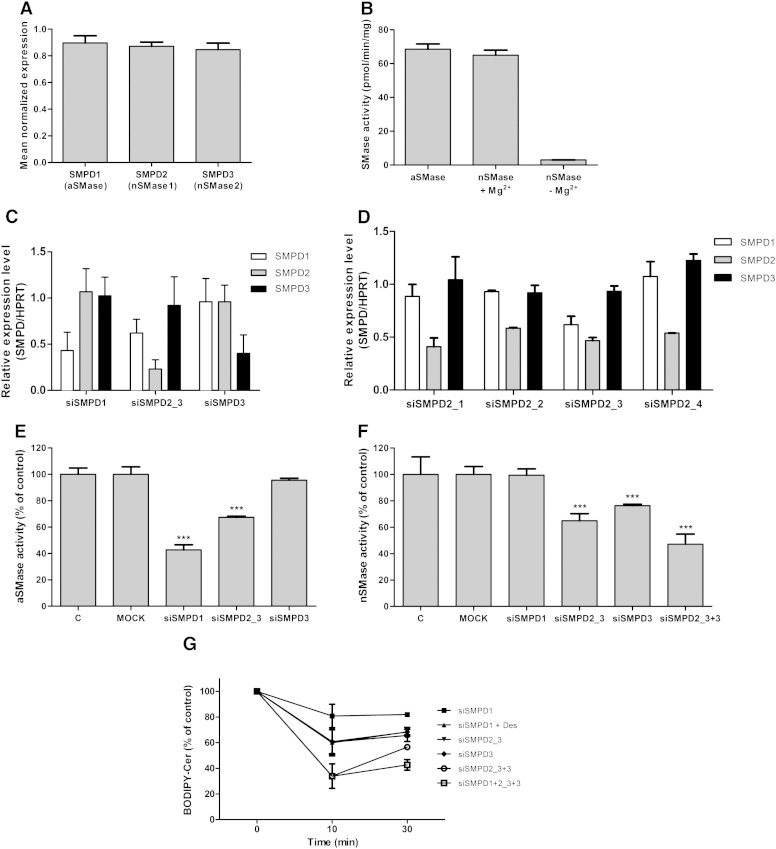
Contribution of individual SMases to BODIPY-SM hydrolysis. (A) SMase expression by CATH.a cells grown under standard conditions was analyzed by RT-qPCR using validated primers for aSMase (SMPD1), nSMase1 (SMPD2), and nSMase2 (SMPD3). Target gene expression is expressed in relation to hypoxanthine-guanine phosphoribosyltransferase (HPRT). Gene expression was calculated by REST analysis as described in Materials and methods. (B) Cells were lysed in lysis buffer under acidic (aSMase activity) or neutral (nSMase activity) conditions and SMase activity of cell lysates was determined as described in Materials and methods. (C) Impact of targeted SMase silencing on transcript expression of nontargeted members. RT-qPCR analyses were performed 48 h post transfection with the indicated siRNAs. Results are expressed as relative expression levels (target gene/HPRT) using REST analysis. (D) Effects of different SMPD2 siRNAs on SMPD1-3 mRNA expression. Silencing was performed with four different 21-mer siRNAs. RT-qPCR analyses were performed 48 h post transfection with the indicated siRNAs. Results are expressed as relative expression levels (target gene/HPRT) using REST analysis. (E, F) Impact of SMase silencing on aSMase (E) and nSMase (F) activity of CATH.a cell lysates. Cells were either untransfected (‘C’), mock transfected, or transfected with the indicated siRNAs. Enzyme activities were determined 48 h post silencing of the indicated target SMase(s). Results represent mean ± SD (n = 3) of one representative experiment. ***p < 0.001 (in comparison to controls). (G) Impact of SMase silencing on BODIPY-SM hydrolysis by intact CATH.a cells. SMase expression was silenced with siRNAs. Forty eight hours post transfection cells were pulse-labeled with BODIPY-SM (1 μM) at 4 °C and chased for 10 and 30 min at 37 °C. Cellular lipids were extracted and BODIPY-SM and BODIPY-Cer concentrations were analyzed by HPLC. Desipramine (Des; 30 μM) treatment of SMPD1-silenced cells was performed for 1.5 h prior BODIPY-SM labeling to enable maximal suppression of BODIPY-SM hydrolysis by aSMase. Results represent the BODIPY-Cer contents in transfected cells normalized to BODIPY-Cer concentrations measured in non-transfected cells (% of controls) and represent mean ± SD (n = 3) of one representative experiment.

Findings shown in Figs. 5–7 demonstrate that endogenous SMases are responsible for SM hydrolysis in CATH.a cells. Expression analysis revealed that the three different SMases, aSMase (gene symbol SMPD1), nSMase1 (SMPD2), and nSMase2 (SMPD3), are present at similar expression levels in CATH.a cells (Fig. 8A). Next we tested whether SMase mRNA expression is reflected on activity level. Indeed we have observed nearly identical aSMase and nSMase activity (BODIPY-SM conversion rates approx. 65 pmol/min/mg cell protein, data in good agreement with another report; [34]). In contrast to aSMase, nSMase activity strongly depends on the presence of Mg^2 +^ (Fig. 8B).

To identify the SMase(s) that are responsible for SM hydrolysis, we applied a siRNA strategy. qPCR analyses of silenced cells revealed downregulation of mRNA levels by 57, 77, and 60% (SMPD1–3; Fig. 8C). While SMPD1 and -3 silencing did not impact on transcript levels of non-targeted SMases, silencing of SMPD2 induced downregulation of SMPD1 by approx. 40%. To address this issue in more detail four different siRNAs were used to silence SMPD2 expression. Results of these analyses (Fig. 8D) revealed that this effect appears to be specific for siSMPD2_3 indicating an off-target effect of this particular siRNA rather than a general counter-regulation of SMPD1 and -3 in response to knockdown of SMPD2.

Silencing of SMPD1 attenuated aSMase activity in cell lysates by 60% and SMPD2 silencing reduced aSMase activity by 37% (Fig. 8E). This off-target effect of siSMPD2_3 on aSMase activity is in line with qPCR data shown in Fig. 8C and D. Silencing of SMPD2 or SMPD3 inhibited nSMase activity by 35 and 25%, respectively. Co-silencing of both nSMases reduced enzyme activity by 55% (Fig. 8F).

Finally we have established the effect of SMase silencing on BODIPY-SM to BODIPY-Cer conversion in intact cells. Forty-eight hours post-transfection cells were pulse-labeled with BODIPY-SM followed by HPLC analysis of BODIPY-SM and BODIPY-Cer of cellular lipid extracts. Knockdown of SMPD1 reduced BODIPY-Cer generation by 20% and this effect was enhanced to 28% in the presence of the lysosomotropic agent Des (Fig. 8G). Knockdown of SMPD2 or SMPD3 or in combination attenuated BODIPY-Cer levels by 25, 33, and 42% after 30 min. A family knockdown including SMPD1–3 reduced cellular BODIPY-Cer concentrations by 68%.

### 3.5 Uptake of HDL-associated BODIPY-SM

**Fig. 9:**
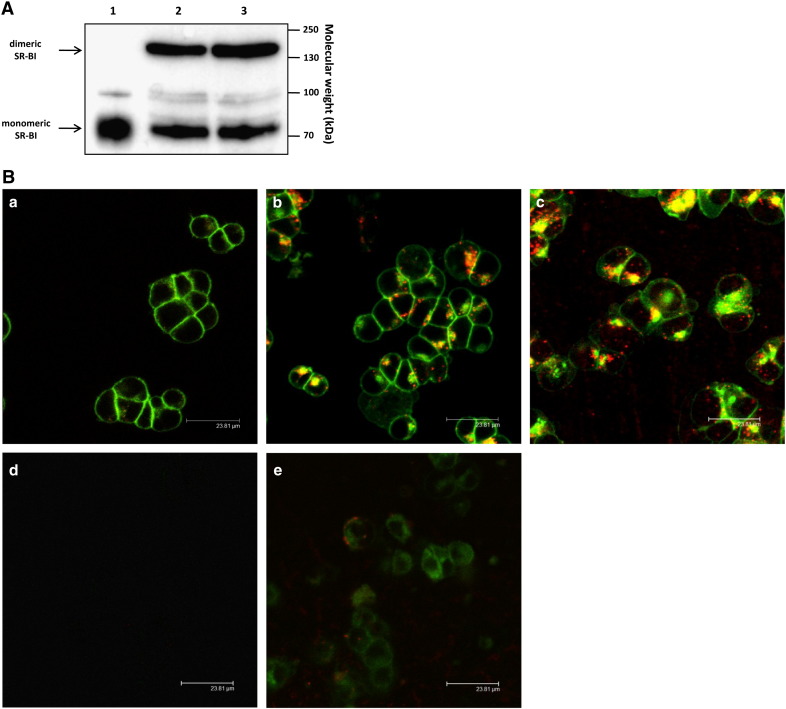

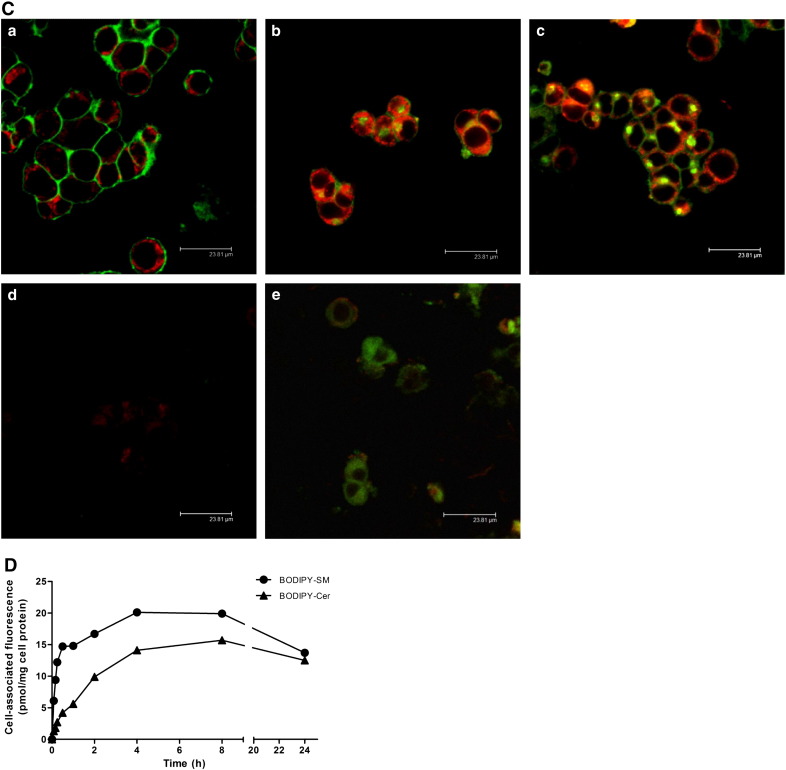
Uptake of HDL-associated BODIPY-SM. (A) Protein lysates obtained from porcine brain microvascular endothelial cells (positive control; lane 1) and CATH.a cells incubated for 24 h in the absence (lane 2) or presence (lane 3) of serum were separated on 10% SDS gels and transferred to PVDF membranes for subsequent detection using anti-SR-BI as a primary antibody. Immunoreactive bands were detected at an apparent molecular weight of approx. 80 and 160 kDa (arrows). (B) Cells were incubated in serum-free medium (a–d) or in the presence of hypertonic sucrose (e) for 30 min followed by addition of BODIPY-SM/DiI-labeled HDL (50 μg HDL protein/ml) for (a) 5 min, (b, d, and e) 60 min, or (c) 240 min at 37 °C. Cells in (d) received a 40-fold excess of unlabeled HDL. After washing with HBSS cells were subjected to LSM analysis. All micrographs were recorded with the same laser intensity. (C) Cells were incubated in serum-free medium (a–d) or in the presence of hypertonic sucrose (e) for 30 min followed by addition of BODIPY-SM/Cy3-labeled HDL (50 μg HDL protein/ml) for (a) 5 min, (b, d, and e) 60 min, or (c) 240 min at 37 °C. Cells in (d) received a 40-fold excess of unlabeled HDL. After washing with HBSS cells were subjected to LSM analysis. All micrographs were recorded with the same laser intensity. (D) Cells were incubated with BODIPY-SM-labeled HDL (50 μg HDL protein/ml) for the indicated times at 37 °C. Cellular lipids were extracted, dissolved in ethanol, and analyzed by HPLC. Results shown represent mean values from one representative experiment performed in duplicates.

In the brain SM is most likely transported in association with apoE/A-I-containing HDL-like particles [35] and HDL-associated lipids are subject to holoparticle and/or selective uptake via initial interaction of apoA-I with SR-BI [36]. First we showed that SR-BI expression levels are not regulated by the serum content of the culture medium (Fig. 9A).

LSM analyses of CATH.a cells incubated with BODIPY-SM (green) and DiI (red; a surrogate tracer for selective uptake of HDL-associated lipids; [37]) -labeled HDL demonstrate that the majority of originally HDL-associated BODIPY-SM remains at the PM at early time points (Fig. 9B, a). At time points ≥ 60 min BODIPY-SM started also to accumulate in intracellular compartments and in some (but not all) cells colocalizes with DiI (yellow; Fig. 9B, b and c). In contrast to BODIPY-SM, DiI staining occurred in a more punctuate staining pattern probably resembling endocytic compartments. An excess of unlabeled HDL efficiently competed for uptake of BODIPY-SM/DiI-labeled HDL (Fig. 9B, d). Pre-treatment with hypertonic sucrose (inducing an endocytic block; [37]) resulted in partial but not complete inhibition of BODIPY- and DiI uptake (Fig. 9B, e). In addition, PM localization of BODIPY-SM changed to diffuse intracellular staining. Taken together, these results indicate that internalization of the majority of fluorescently-labeled HDL is mediated apparently by SR-BI-dependent endocytosis.

HDL covalently labeled with Cy3 in the protein moiety showed a more pronounced staining (Fig. 9C) as compared to DiI-labeled HDL. Of note, Cy3-labeled HDL uptake is a fast process with tracer accumulation in intracellular compartments already visible after 5 min (Fig. 9C, a). Whereas intracellular localization of Cy3 fluorescence remained more or less unaffected during the entire incubation period, BODIPY-SM localized almost exclusively to the PM after a 5 min chase at 37 °C (Fig. 9C, a) and started to accumulate in perinuclear regions at time points ≥ 60 min where it partially colocalizes with the Cy3-labeled HDL protein moiety (Fig. 9C, b and c). Both an excess of unlabeled HDL and hypertonic sucrose treatment strongly reduced BODIPY-SM and Cy3 staining (Fig. 9C, d and e). Taken together, these results indicate that BODIPY-SM uptake is less affected by hypertonic sucrose as compared to DiI- and Cy3-HDL uptake.

Quantitation of BODIPY-SL in CATH.a cells incubated with BODIPY-SM-labeled HDL revealed saturable uptake kinetics. Cell-associated SM levels reached a maximum concentration of 20 pmol/mg cell protein (4 h; Fig. 9D). However, compared to carrier-free BODIPY-SM, hydrolysis occurred at slower rates and the levels of generated BODIPY-Cer did not exceed that of BODIPY-SM.

## 4 Discussion

Here we present evidence that internalization of BODIPY-SM by the murine CATH.a neuronal cell line is energy-dependent and primarily mediated by caveolar mechanisms. BODIPY-SM (or BODIPY-Cer generated from the probe) is not delivered to lysosomes but is transported to the ER/Golgi complex, probably via recycling endosomes. Cer generation from SM is more efficient than SM synthesis from Cer indicating high SMase and lower SMS activity. Since a major fraction of SM resides in the PM it is conceivable that SMase-mediated hydrolysis of BODIPY-SM starts already in this compartment. Pharmacological inhibition and RNAi experiments suggest that both aSMase and nSMases contribute to SM hydrolysis.

Steady state and pulse-chase labeling experiments revealed that at 4 °C BODIPY-SM accumulates in the PM of CATH.a neurons. A switch to 37 °C results in the occurrence of fluorescent punctuate structures indicating vesicle mediated transport with the tracer finally ending up in the Golgi (Fig. 1). This parallels previous studies where fluorescently-labeled SM analogs were observed in these compartments after a short incubation period at 37 °C [26,38,39]. Babia et al. [40] demonstrated that internalization of NBD-SM was energy- and temperature-dependent, and that intracellular transport was insensitive to the NBD-fluorescence quencher sodium dithionite. These results indicate that vesicles mediate the transport of internalized NBD-glucosylceramide (NBD-GlcCer) and NBD-sphingomyelin to the Golgi complex [40]. The same authors have demonstrated that only a small fraction of NBD-SM in the PM is subjected to hydrolysis to Cer while internalized NBD-SM remained unaltered in primary rat hippocampal neurons. This contrasts with our findings in the murine CATH.a cell line, where a substantial fraction of BODIPY-SM was hydrolyzed to BODIPY-Cer independent whether a steady state or pulse-chase labeling protocol was used (Figs. 5 and 6). Therefore it is likely that BODIPY-Cer provides a significant contribution to Golgi fluorescence (Fig. 2). In addition to the Golgi we have also observed accumulation of BODIPY-tagged lipids in the ER. Galactosylceramide (GalCer) and, unlike to other cells, GlcCer synthesis in neurons takes place in the ER [41]. This raises the possibility that BODIPY-Cer generated via SMase activity reaches the ER, and serves as precursor for GlcCer and/or GalCer synthesis. However, these issues were not addressed during the present study. In line with other groups [39,42] we have been unable to detect fluorescently-labeled SM in lysosomes.

There is limited information on the mechanisms of SM internalization from the PM and subsequent intracellular targeting. Most of the data are based on studies with fluorescently-labeled SM. NBD-SM incorporated in the PM is endocytosed and rapidly sorted for recycling to the PM [43] whereas BODIPY-SM predominantly labels the Golgi [26]. Golgi labeling may be due to vesicular transport of BODIPY-SM from endosomes to the Golgi and/or hydrolysis of BODIPY-SM to the corresponding BODIPY-Cer, which has a high affinity for the Golgi [44]. Endocytic trafficking of PYRENE-SM depends on the acyl chain length [38]: short-chain SM recycle more effectively to the PM while long-chain SM are transported along the late endocytic pathway and recycle in an NPC1- and cholesterol-dependent manner. Puri et al. [26] have demonstrated that different SL are internalized via different endocytic pathways. While lactosylceramide (LacCer) and globoside are internalized almost exclusively via caveolar pathways, BODIPY-SM uptake was mediated to equal parts by clathrin- and caveolae-dependent pathways [26]. Caveolar SL internalization in fibroblasts occurs along microtubules (as observed during the present study; Fig. 2D, b) and depends on phosphoinositol 3-kinases and rab proteins [45]. To elucidate uptake mechanisms for BODIPY-SM in CATH.a cells inhibitors of clathrin- and caveolae-mediated endocytosis were tested (Figs. 3 and 4). Pronounced inhibition of BODIPY-SM uptake by antagonists of the caveolar pathway strongly suggests that this is quantitatively the more important route in CATH.a neurons. Endocytic pathways of fluorescently-labeled SM analogs are somewhat controversial. Whereas some groups suggested SM uptake in clathrin-coated pits and caveolae [26], other studies highlight SM stereochemistry as a major determinant which endocytic pathway is chosen: Naturally occurring BODIPY-D-*erythro*-SM (as used during the present study) is internalized via a NY-sensitive, CP-insensitive mechanism in human skin fibroblasts, whereas BODIPY-L-*threo*-SM is taken up via a CP-sensitive mechanism, similar to what was observed with stereoisomers of BODIPY-labeled LacCer [46,47].

Alternatively, clathrin- and caveolin-independent membrane endocytotic processes have been reported: Zha et al. [48] demonstrated that treatment of ATP-depleted macrophages and fibroblasts with exogenous SMase rapidly induces formation of numerous vesicles that pinch off from the PM. These authors [48] concluded that hydrolysis of SM on the PM causes inward curvature and subsequent fusion to form sealed vesicles that lack a caveolin or clathrin coat. These findings suggest that (at least some) mammalian cells use SMase to change lipid composition locally thereby promoting membrane budding and fusion.

Of note, comparable observations were reported for SMase-treated protein-free giant vesicles composed of PC, SM, and BODIPY-SM [49,50]. The authors demonstrated that SMase added to the outside of SM-containing giant vesicles results in an endocytosis-like process solely dependent on newly formed Cer-enriched microdomains [49]. This process involves SMase-mediated Cer formation in the outer leaflet, lateral phase separation of Cer-enriched domains, invagination, budding, and finally ‘endocytosis’ of these vesicles into the giant vesicles. In contrast, microinjection of SMase into the interior of giant vesicles (leading to SM hydrolysis at the inner leaflet) resulted in liposome formation on the outer leaflet [49]. Collectively these findings suggest that membrane endocytosis or blebbing (depending on whether Cer is formed at the outer or inner leaflet of the membrane) might be driven by Cer formation independent of metabolic energy and a caveolin- or clathrin coat.

Steady state labeling of CATH.a cells revealed lower uptake kinetics (probably due to the lack of the polar phosphocholine headgroup) of BODIPY-Cer compared to BODIPY-SM (Fig. 5). Also PYRENE-SM uptake was slower as compared to the corresponding BODIPY analog most probably a result of the higher hydrophobicity of the PYRENE tag. These results indicate different, fluorophore-dependent uptake properties (see also above) of tagged lipids and their specific (dis)advantages with regard to partitioning into ordered domains of the PM have been addressed [51,52].

Of note, SMases display similar enzyme affinity for NBD- or BODIPY-SM as compared to their natural counterparts [24,53]. Our pulse-chase studies (Fig. 7) demonstrated that BODIPY-SM uptake and metabolic conversion of the tracer occur rapidly. The preference of neurons for SM hydrolysis over SM synthesis is supported by studies that showed rapid NBD-SM hydrolysis in neurons and slow conversion of NBD-Cer to NBD-SM while the opposite is true for oligodendrocytes [54]. Although increased intracellular Cer concentrations are considered pro-apoptotic [55], SMase-mediated Cer synthesis could fuel a ceramidase-dependent [56], Sph-generating pathway that is centrally involved in SNARE assembly and synaptic vesicle fusion [21,22]. A significant fraction of BODIPY-SM was already metabolized after pulse labeling at 4 °C. This suggests that SMases are enzymatically active even at low temperatures, which is in line with others [57,58]. Interestingly a quantitatively significant proportion of BODIPY-SM and BODIPY-Cer as well as an unidentified BODIPY-SL were recovered from the cellular supernatant (Fig. 6B). Using two different experimental approaches (Fig. 7) we could show that a major fraction of exogenously supplied BODIPY-SM is localized in the PM where it would be accessible to SMases. Whether the presence of BODIPY-labeled SL in the supernatant is a result of synthesis/release from the outer leaflet of the PM or due to active efflux is currently under investigation.

Using siRNAs and pharmacological inhibition our results suggest that aSMase as well as nSMases contribute to BODIPY-SM hydrolysis (Fig. 8). This is in line with our observation of almost identical activities of aSMase and Mg^2 +^-dependent nSMase in CATH.a cell lysates. Despite the absence of BODIPY-SM colocalization with the LysoTracker (Fig. 2C) BODIPY-SM hydrolysis could occur at the PM, where a substantial proportion of aSMase is localized [59–61]. Also nSMase(s) have been shown to localize at the PM and palmitoylation was reported to facilitate nSMase2 localization at the cytosolic face of the PM [62]. Whether nSMase-mediated hydrolysis of SM at the PM (as shown for exogenously added nSMase in hippocampal neurons; [63]) contributes to the generation of S1P at the PM remains to be elucidated. Studies in PC12 cells demonstrated localization of nSMase to the Golgi [64], supporting the idea that part of BODIPY-SM is hydrolyzed at this organelle. Additionally, recent studies have shown that nSMase can translocate from the PM or the Golgi to early endosomes [33]. These findings implicate that nSMase activity could contribute to BODIPY-SM hydrolysis in several subcellular compartments.

Findings of the present study indicate that HDL could act as physiological carrier and donor of SM. HDL lipids are taken up by cells/tissues either through HDL holoparticle internalization or endocytosis-dependent [65] or -independent [37] selective uptake via SR-BI. Along this line it is important that selective uptake of HDL-associated SM is mediated by SR-BI and depends on intact caveolae [66]. In the CNS delivery of SM via apoE-containing, reconstituted HDL particles stimulates axonal extension, indicating a potential involvement of HDL-associated SM in nerve regeneration [67]. The authors of that study [67] also demonstrated that this process requires LDL receptor-related protein-1 indicating that this receptor mediates neuronal holoparticle uptake of apoE-containing lipoproteins.

On basis of competition and hypertonic sucrose inhibition experiments the present study indicates that BODIPY-SM uptake occurs primarily via endocytic pathways. In line, HDL particles were shown to undergo endocytosis, lose lipids, followed by resecretion of lipid-depleted particles by non-polarized hepatocytes [68]. In polarized hepatocytes HDL particles enter the endocytic recycling compartment paralleling intracellular trafficking of SR-BI [65]. Data obtained during the present study where sucrose inhibited the uptake of all fluorescent, HDL-associated tracers (independent whether bound covalently or non-covalently) would support an uptake mechanism of HDL-associated BODIPY-SM that is coupled to endocytosis. However, it was also demonstrated that selective uptake of HDL-associated lipids occurs independently of HDL holoparticle endocytosis [37]. It is unlikely that the difference of cellular uptake of DiI- and Cy3-labeled particles is due to major alterations in HDL surface charge since labeled and unlabeled HDL comigrated on agarose gels (data not shown).

Taken together we demonstrate that fluorescent SL analogs are rapidly metabolized in CATH.a cells. BODIPY-SM uptake occurs predominately via caveolar pathways and is subject to hydrolysis already at the PM, in line with results reported for NBD-SM [58]. The activity of aSMase and nSMase at the PM generates BODIPY-Cer that reaches intracellular compartments leading to Golgi and ER accumulation [39]. ER/Golgi localization of BODIPY-Cer results in generation of polar SL that are secreted into the cellular supernatant. Most of the residual SM resides at the PM whereas the remaining part is internalized to intracellular compartments via early endosomes (as shown for NBD-SM; [69]) or is transported back to the PM via recycling endosomes. Endocytosed BODIPY-SM is transported to intracellular compartments of SL synthesis (ER and Golgi) bypassing lysosomal degradation. This is in agreement with other studies that showed that PYRENE- and NBD-labeled short-chain SM analogs efficiently recycle to the PM after endocytosis and can directly target the Golgi [38,58]. Our observation that HDL-associated BODIPY-SM is efficiently taken up by neurons might be of particular importance for SM homeostasis in the CNS.

## Figures and Tables

**Table 1:** Solvent gradient used for elution of BODIPY-labeled lipids.

Time [min]	Flow rate [ml/min]	Solvent A^a^ [%]	Solvent B^b^ [%]	Solvent C^c^ [%]
5.0	0.7	80	20	0
8.0	0.7	0	100	0
11.7	0.7	0	100	0
12.0	1.0	0	100	0
15.0	1.0	0	20	80
20.0	1.0	0	20	80
20.5	1.0	80	20	0
23.0	0.7	80	20	0

a Solvent A: MeOH:H_2_O:Tris/HCl (0.5 M, pH 9), 93:4:3 (v/v/v).

b Solvent B: Acetonitrile.

c Solvent C: MeOH:Tris/HCl (0.5 M, pH 9), 97:3 (v/v).

**Table 2:** Solvent gradient used for elution of PYRENE-labeled lipids.

Time [min]	Flow rate [ml/min]	Solvent A^a^ [%]	Solvent B^b^ [%]	Solvent C^c^ [%]
5.0	0.7	0	20	80
6.0	0.7	0	100	0
7.7	0.7	0	100	0
8.0	1.0	0	100	0
10.0	1.0	40	20	40
18.5	1.0	40	20	40
21.0	0.7	40	20	40
33.0	0.7	40	20	40
33.5	0.7	0	20	80
36.0	0.7	0	20	80

a Solvent A: MeOH:Tris/HCl (0.5 M, pH 9), 99:1 (v/v).

b Solvent B: Acetonitrile.

c Solvent C: MeOH:Tris/HCl (0.5 M, pH 9), 97:3 (v/v).
